# A 6-year follow-up study in a community-based population: Is neighbourhood-level social capital associated with the risk of emergence and persistence of psychotic experiences and transition to psychotic disorder?

**DOI:** 10.1017/S0033291722000642

**Published:** 2023-07

**Authors:** Ceylan Ergül, Marjan Drukker, Tolga Binbay, Umut Kırlı, Hayriye Elbi, Köksal Alptekin, Jim van Os

**Affiliations:** 1 Department of Psychiatry, Faculty of Medicine, Üsküdar University, Istanbul, Turkey; 2 Department of Psychiatry and Neuropsychology, School for Mental Health and Neuroscience, Maastricht University Medical Center, Maastricht, The Netherlands; 3 Department of Psychiatry, Faculty of Medicine, Dokuz Eylul University, Izmir, Turkey; 4 Institute on Drug Abuse, Toxicology and Pharmaceutical Science, Ege University, Izmir, Turkey; 5 Department of Psychiatry, Faculty of Medicine, Ege University, Izmir, Turkey; 6 Department of Psychiatry, UMC Utrecht Brain Centre, University Medical Centre Utrecht, Utrecht University, Utrecht, The Netherlands; 7 Department of Psychosis Studies, Institute of Psychiatry, Psychology & Neuroscience, King's College London, London, UK

**Keywords:** neighbourhood, psychosis, psychotic disorder, psychotic experiences, social capital, social disorganisation

## Abstract

**Background:**

Social capital is thought to represent an environmental factor associated with the risk of psychotic disorder (PD). This study aims to investigate the association between neighbourhood-level social capital and clinical transitions within the spectrum of psychosis.

**Methods:**

In total, 2175 participants, representative of a community-based population, were assessed twice (6 years apart) to determine their position within an extended psychosis spectrum: no symptoms, subclinical psychotic experiences (PE), clinical PE, PD. A variable representing change between baseline (T1) and follow-up (T2) assessment was constructed. Four dimensions of social capital (informal social control, social disorganisation, social cohesion and trust, cognitive social capital) were assessed at baseline in an independent sample, and the measures were aggregated to the neighbourhood level. Associations between the variable representing psychosis spectrum change from T1 to T2 and the social capital variables were investigated.

**Results:**

Lower levels of neighbourhood-level social disorganisation, meaning higher levels of social capital, reduced the risk of clinical PE onset (OR 0.300; *z* = −2.75; *p* = 0.006), persistence of clinical PE (OR 0.314; *z* = −2.36; *p* = 0.018) and also the transition to PD (OR 0.136; *z* = −2.12; *p* = 0.034). The other social capital variables were not associated with changes from T1 to T2.

**Conclusions:**

Neighbourhood-level social disorganisation may be associated with the risk of psychosis expression. Whilst replication of this finding is required, it may point to level of social disorganisation as a public health target moderating population psychosis risk.

## Introduction

Social capital may represent an environmental factor influencing the geographical distribution of psychotic disorder (PD; Krabbendam & Van Os, 2005). The dynamic changes within the spectrum of psychosis expression, or extended psychosis phenotype may yield valuable implications. Here, we examine prospective associations between neighbourhood-level social capital and changes within the extended psychosis phenotype over time.

Social capital is generally used to describe the aspects of social networks, relations and trust either at the individual or area level (Whitley & McKenzie, 2005). It can be seen as an umbrella term including informal social control, social cohesion and trust, social disorganisation and cognitive social capital (Moore & Kawachi, 2017). Informal social control is the willingness of the group to enforce social norms for the common good (Kawachi & Berkman, 2015). Social cohesion and trust refers to the extent of connectedness and solidarity among individuals in a society (Kawachi & Berkman, 2015). Social disorganisation is defined as a disturbance in the patterns and mechanisms of human relations (Faris & Dunham, 1939). Cognitive social capital refers to people's perceptions of trust, support and reciprocity (Harpham, Grant, & Thomas, 2002). Higher social capital has been associated with better physical and mental health outcomes (Ehsan, Klaas, Bastianen, & Spini, 2019). In 1939, a higher schizophrenia prevalence was observed in urban neighbourhoods with a higher level of social disorganisation (Faris & Dunham, 1939). Afterwards, it was claimed that one-third of schizophrenia incidence was associated with environmental factors, and social capital has been hypothesised as a possible underlying environmental factor mediating the impact of urbanicity on the non-random geographical distribution of PD (Krabbendam & Van Os, 2005).

Psychosis has been placed on a spectrum of severity, including PD and non-psychotic diagnoses in clinical populations and psychotic experiences (PE) in the general population, forming an extended psychosis phenotype (Kaymaz & Van Os, 2010; Linscott & Van Os, 2013; Van Os & Linscott, 2012). Findings indicate demographic (age), psychopathological (negative symptoms, cognitive alterations), genetic, environmental (childhood adversity, urbanicity) and temporal continuity between PE and PD (Linscott & Van Os, 2010; Stip & Letourneau, 2009; Van Os, Linscott, Myin-Germeys, Delespaul, & Krabbendam, 2009). Even though most PE recover spontaneously (Zammit et al., 2013), they increase the risk of developing PD later (Hanssen, Bak, Bijl, Vollebergh, & van Os, 2005). Eighty per cent of PE are transitory, 20% are persistent and 7% change to a PD over time (Linscott & Van Os, 2013; Zammit et al., 2013). Factors predicting transition have been investigated in several longitudinal studies (Kaymaz & Van Os, 2010). The results suggest that number (Poulton et al., 2000) and rate of persistence of PE (Dominguez, Wichers, Lieb, Wittchen, & Van Os, 2011), type of coping (Bak et al., 2003) as well as the presence of affective dysregulation (Van Rossum, Dominguez, Lieb, Wittchen, & Van Os, 2011) and negative symptoms (Dominguez, Saka, Lieb, Wittchen, & Van Os, 2010) affect the likelihood of transition.

A recent review studying the association between social capital and psychosis reported conflicting results as a consequence of heterogeneous measures of social capital (Rotenberg, Anderson, & McKenzie, 2020). An earlier study did not find any association between social capital and schizophrenia incidence but did show that the quantity of inpatient service consumption was higher in neighbourhoods with a higher level of informal social control (Drukker, Krabbendam, Driessen, & van Os, 2006). On the other hand, two studies, including large cohorts, concluded that low levels of social capital were associated with a higher incidence of PD. Kirkbride et al. suggested that the association between social capital and schizophrenia was not linear but U-shaped; incidence rates being higher in neighbourhoods with both low and high levels of social capital but not median levels of social capital (Kirkbride et al., 2008). In a non-clinical population, paranoia was associated with the measures of social capital, including decreased trust in others (Freeman et al., 2011). In that study, data regarding symptoms of psychosis and social capital were collected from the same population, raising the possibility of contamination of the social capital data with the individual perceptions of the study population (Drukker & van Os, 2003). In conclusion, there is some evidence that the measures of social capital, including disorganisation and control, are associated with psychosis (Rotenberg et al., 2020).

The present paper is a part of The Izmir Mental Health Survey for Gene-Environment in Psychoses (TürkSch). The first wave of the TürkSch study confirmed the continuity of PE and PD and revealed that 25% of the general population has experiences that phenotypically are in the extended psychosis spectrum (Binbay et al., 2012c). Baseline data showed that as neighbourhood-level social capital (informal social control) and social deprivation increased, the expression of psychosis moved towards the clinical/syndromal end of the spectrum. Furthermore, the association between familial liability of severe mental illness and expression of psychosis was stronger in neighbourhoods with higher levels of deprivation and social control (Binbay et al., 2012b). This finding contradicts the results of studies from Maastricht and London, where the expression of psychosis was more at the clinical/syndromal end in neighbourhoods with lower levels of social capital (Drukker et al., 2006; Kirkbride et al., 2007). The contradiction may be attributed to the higher mean neighbourhood-level social control in Izmir (Dominguez et al., 2010; Lofors & Sundquist, 2007). The finding thus suggests that the effect of social capital on the expression of psychosis is not linear.

As summarised above, the current literature provides conflicting results regarding the association between social capital and the expression of psychosis in cross-sectional designs. As far as we know, this is the first longitudinal study prospectively investigating the role of social capital in occasioning transitions across the severity spectrum of the extended psychosis phenotype. This paper aims to assess whether neighbourhood-level social capital is associated with the risk of psychosis transitions across a spectrum of severity, ranging from no PE, subclinical PE, clinical PE to PD, from baseline (T1) to 6-year follow-up (T2) in a general population sample. More specifically, we assessed whether:

1. baseline characteristics were associated with any of the dynamic transitions from T1 to T2 and specifically with emergence, persistence and transition.
2. social capital is associated with the risk of any transition from T1 to T2.
3. social capital is associated with the risk of three predefined transitions: emergence of PE, persistence of PE, transition to PD.

## Methods

### Study design and data collection

TürkSch is a prospective, longitudinal study designed to screen and follow up the extended psychosis phenotype and individual, family and neighbourhood-level variables (Binbay et al., 2011). Data were collected in Izmir, which is the third most urbanised area in Turkey (Türkiye İstatistik Kurumu, 2008). TürkSch consisted of a baseline (T1: 2007–2009) and a follow-up assessment (T2: 2013–2015), approximately 6 years apart (mean follow-up time: 6.11 ± 0.94 years). Each assessment consisted of several stages of data collection (T1: stages 1–3; T2: stages 4–5). The present paper used data collected at stages 1, 2 and 4. At stage 1, a baseline sample was randomly selected representing the wider Izmir metropolitan area using a multistage sampling procedure, stratified by urbanicity covering 11 districts and 302 neighbourhoods. The first stage was a cross-sectional study, including assessment of the extended psychosis spectrum and individual-level sociodemographic variables (T1, stage 1, *n* = 4011). For stage 2, a second address from the same neighbourhood was contacted for each participant of stage 1. Thus, a different sample from the same area provided neighbourhood-level social capital variables (T1, stage 2, *n* = 5819). Finally, 6 years after baseline, addresses of all T1 participants were revisited in person. The extended psychosis spectrum psychopathology was collected as well as changes in sociodemographic features, alcohol and cannabis use, and threatening life events from all stage 1 participants who could be reached (T2, stage 4, *n* = 2185). Details of stages 1–5 have been described elsewhere (Binbay et al., 2011, 2012a; Kırlı et al., 2019). The study was approved by the ethics committee of Ege University; written informed consent was obtained from all participants.

### Assessment of the sociodemographic features

The T1 assessment included a sociodemographic questionnaire. At T2, the same questionnaire was used to determine changes over time. Level of education was dichotomised into ⩽8 and >8 years; ethnicity into Turkish and non-Turkish; marital status into married and non-married; employment status into unemployed and not unemployed; alcohol and cannabis use into user and non-user; migration between ages 6 and 15 years into migrated *v.* not migrated; childhood adversity and traumatic events into none *v.* at-least once. Childhood adverse life events were the death of any parent, divorce of parents and separation from parents for at least 3 months between the age of 0 and 15 years. Traumatic events were war experience, life-threatening accident, fire, natural disasters, witnessing someone being badly injured or killed, rape, sexual molestation and being physically attacked or assaulted. Socioeconomic status was based on the participant's profession, recoded into four ordinal categories: professional and non-manual high employees; non-manual low employees and skilled workers and technicians; owners of small businesses; manual workers. In order to estimate socioeconomic status at birth, parental socioeconomic status was coded similarly, using the highest position of the participant's mother or father. The data included three urbanicity variables: urbanicity at birth, urbanicity at age 6–15 years and current urbanicity. Urbanicity at birth and in childhood was based on population density measures in the 1990 census, and the classification depended on the level of organised features of streets and buildings. If the participant lived at more than one address, the most urban one was included. Current urbanicity was coded into four categories: rural, low-urban, moderately-urban, highly-urban area. Family history of mental disorder was recoded into four categories (none, other/unknown, common mental disorder, severe mental disorder). Common mental disorder in the family included any diagnosis of depression, anxiety, conversion or somatisation in the absence of severe mental disorder among parents or siblings of the participant. Severe mental disorder in the family was coded positive if any of the participant's first-degree relatives had been diagnosed with PD or bipolar disorder, had died because of suicide or had been admitted to a psychiatric inpatient unit.

### Assessment of the social capital of neighbourhoods

Four dimensions of neighbourhood-level social capital were assessed at stage 2: informal social control, social cohesion and trust, social disorganisation, and cognitive social capital. The *informal social control* scale included eight questions measuring the willingness to intervene in hypothetical neighbourhood-threatening situations such as children misbehaving, using a five-point Likert scale ranging from ‘strongly disagree’ to ‘strongly agree’. The questions were derived from the Sampson collective efficacy scale and adapted for use in the Turkish population (Sampson, Raudenbush, & Earls, 1997). The *social cohesion and trust* scale measured bonds and trust among neighbourhood residents (Araya et al., 2006; Sampson et al., 1997). The *social disorganisation* scale consisted of eight questions rating the frequency of certain scenarios occurring in the participant's neighbourhood, such as the presence of graffiti, vandalism, burglary and racist attacks. Items were assessed using a four-point Likert scale ranging from ‘not at all common’ to ‘very common’, and the scale was derived from the McCulloch instrument (Buckner, 1988; McCulloch, 2003). The *cognitive social capital* scale included three questions measuring perceptions of support, reciprocity and sharing among the residents of the neighbourhood (Harpham et al., 2002).

For each social capital dimension, sum scores were (negative items were reversed) divided by the number of items and aggregated to the neighbourhood level. Lower scores on the informal social control variable (ISC-variable) indicated higher levels of informal social control, meaning higher social capital. Similarly, lower scores on the social cohesion and trust variable (SCT-variable) indicated higher levels of social cohesion and trust, meaning higher social capital. On the other hand, lower scores on the social disorganisation variable (SocD-variable) indicated higher levels of social disorganisation, meaning lower social capital. Similarly, lower scores on the cognitive social capital variable (Cogn-variable) indicated lower levels of cognitive social capital, meaning lower social capital (Binbay et al., 2012b). All four social capital variables were significantly correlated with each other. The correlation coefficients varied; ISC-variable, SCT-variable and Cogn-variable were moderately to strongly correlated with each other, whereas the SocD-variable was weakly correlated with the others (Table 1).

**Table 1.** Spearman's rank order correlation values between the four social capital variables (*p* < 0.001 for all correlations)

	ISC-variable	SCT-variable	Cogn-variable	SocD-variable
ISC-variable	1.000			
SCT-variable	0.593	1.000		
Cogn-variable	−0.511	−0.790	1.000	
SocD-variable	−0.256	−0.259	0.227	1.000

ISC-variable, informal social control variable. Higher scores on the ISC-variable indicate lower levels of informal social control, meaning lower social capital.

SCT-variable, social control and trust variable. Higher scores on the SCT-variable indicate lower levels of social control and trust, meaning lower social capital.

SocD-variable, social disorganisation variable. Higher scores on the SocD-variable indicate lower levels of social disorganisation, meaning higher social capital.

Cogn-variable, cognitive social capital variable. Higher scores on the Cogn-variable indicate higher levels of cognitive social capital, meaning higher social capital.

### Assessment of the extended psychosis spectrum

The procedure used to identify PE and PD has been described in detail elsewhere (Binbay et al., 2011). The assessed time frame was lifetime at T1, whereas it was the last 6 years at T2. Hallucinations, delusions, and associated impairment were assessed using the relevant sections of the Turkish version of the Composite International Diagnostic Interview (CIDI) 2.1 (Kılıç & Göğüş, 1997). The CIDI is a fully structured interview that can be used by both clinicians and trained interviewers (Andrews & Peters, 1998). It was designed by the WHO to be implemented in epidemiological studies of mental disorders, and it provides information about frequency, duration and severity of symptoms, psychosocial impairment and help-seeking (Robins et al., 1988). It enables diagnosing various mental disorders in accordance with the criteria of the ICD-10 (World Health Organisation, 1992) and DSM-IV (American Psychiatric Association, 1994). CIDI has excellent inter-rater reliability in almost all sections, with *κ* values ranging from 0.67 to 0.97 (Wittchen et al., 1991). The *κ* value for agreement between clinicians for delusions and hallucinations was 0.85 and 0.87, respectively. Sensitivity of the CIDI was higher than its specificity for both delusions (0.93 *v.* 0.55) and hallucinations (0.86 *v.* 0.50) (Cooper, Peters, & Andrews, 1998).

Additionally, if the participant had ever been diagnosed with a mental disorder or been treated for a mental health problem, the health professional involved in diagnosis or treatment was contacted to gather relevant information when needed. If a participant had never been diagnosed with a PD but endorsed at least one CIDI psychotic symptom associated with help-seeking or occurring with a frequency of at least once per week, the participant was invited for a clinical evaluation. When the participant did not show up at the hospital visit, clinical interviews were conducted by the psychiatrist either at the participant's residence or via telephone. If clinical reappraisal was insufficient to get a final diagnosis, two further steps were applied with the participant's written consent. First, a family member was called to ask further questions on the illness and treatment of the probable case. Second, the patient register databases of the two university hospitals (Ege and Dokuz Eylül University Hospitals) in Izmir were checked. A best-estimate diagnosis of the CIDI interview was made in a collective evaluation according to the Structured Clinical Interview for DSM-IV Axis I Disorders (SCID-I) (First, 1997).

Finally, an extended psychosis phenotype variable was constructed based on the relevant CIDI items, clinical registries and the SCID-I results. The variable consisted of four categories:

1. *No PE*: No positive rating on any of the items of the CIDI psychosis section.
2. *Subclinical PE*: Presence of PE not leading to any distress, impairment or help-seeking.
3. *Clinical PE*: Presence of PE and positive score on any of the seven CIDI impairment items in the absence of a diagnosis of a PD.
4. *PD*: Current or past DSM-IV diagnosis of any disorder with psychotic features, based on either clinical registries or evaluation by the clinician as a part of the TürkSch study.

### Dependent variable

**Fig. 1.**
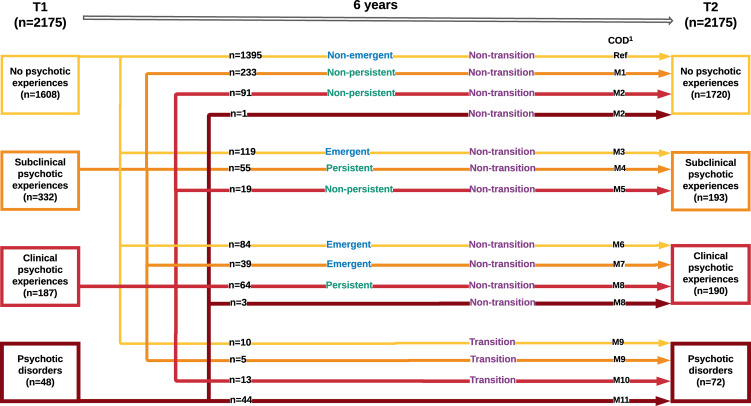
Dynamic transitions over time in the extended psychosis spectrum and groups defined for analysis. COD, categories of the dependent variable.

The dependent variable was constructed using the extended psychosis phenotype variable of all the participants who were interviewed at both T1 and T2 and who were residing in neighbourhoods with social capital data (*N* = 2175). Figure 1 shows all possible combinations of values at T1 and T2.

For the purpose of the analyses, three groups were defined as follows:

*Emergent group*: Those who had no PE at T1 but had subclinical (*n* = 119) or clinical (*n* = 84) PE at T2, as well as those who had subclinical PE at T1 and clinical PE at T2 (*n* = 39), yielding a total of 242.

*Persistent group*: Those who had either subclinical PE at both T1 and T2 (*n* = 55) or clinical PE at T1 and T2 (*n* = 64), yielding a total of 119.

*Transition group*: Those who had no PE (*n* = 10) or some subclinical (*n* = 5) or clinical (*n* = 13) PE at T1 and PD at T2, yielding a total of 28.

### Statistical analyses

All analyses were performed using Stata, version 13.1 (StataCorp, 2013). In the present data, social capital was a characteristic at the neighbourhood level. Thus, data had a multilevel structure with subjects clustered in neighbourhoods. All statistical analyses were, therefore, performed with the equivalent multilevel format of the specific command, thus taking into account the hierarchical structure of the data.

In order to investigate whether neighbourhood-level social capital is associated with dynamic transitions within different levels of the extended psychosis phenotype over the follow-up period (T1 to T2), a multinomial logistic regression analysis was performed, including all 2175 cases. The first model included age, gender and parental socioeconomic status. Unfortunately, the multinomial analysis did not reach convergence and this forced us to remove parental socioeconomic status. The four neighbourhood-level social capital variables were added in separate models to avoid collinearity. Participants without expression of psychosis at any level at both T1 and T2 were used as the reference group. Three groups consisting of ≤5 participants were combined with the group above, resulting in 11 groups to be compared (labelled M1–11) with the reference group (Fig. 1).

Then, emergence, persistence and transition outcomes were analysed separately:

1. Emergence of PE: emergent (*n* = 242) (M3, M6–7) *v.* non-emergent (*n* = 1395) (Ref)
2. Persistence of PE: persistent (*n* = 119) (M4, M8) *v.* non-persistent (*n* = 343) (M1–2, M5)
3. Transition to PD: transition (*n* = 28) (M9–10) *v.* non-transition (*n* = 2103) (Ref, M1–8)

The baseline sociodemographic characteristics of the three outcome groups were compared using χ^2^ test. Mixed-effects logistic regression analysis was performed with neighbourhood-level social capital as the macro-level independent variable and emergence, persistence and transition as the three dependent variables. First, a model was analysed, including gender, age categories and parental socioeconomic status (model 1). Following this, each of the four neighbourhood-level social capital variables was added to model 1 in separate models.

Additional analyses were performed comparing the social capital data of the participants who moved to another neighbourhood during the 6-year follow-up with the ones who did not move in order to examine if this is also relevant.

## Results

At T1, the majority of the 2175 participants were female (59.4%), married (70.4%), of Turkish ethnicity (72.1%), and educated for ⩽8 years (60.5%). The mean age of the participants was 38.6 ± 13.3 (15–65). The proportion of participants who used alcohol was 45.4, and 2.9% were cannabis users.

### Any transitions over time within the extended psychosis phenotype

Multinomial logistic regression analysis revealed that women recovered more frequently from clinical PE (M2; Table 2). Older people were less likely to have emergent subclinical PE (M3). However, if they did, they were less likely to recover (M1). Having persistent clinical PE was less likely for older people (M8), who were also less likely to transition to PD (M10).

**Table 2.** Multinomial logistic regression analysis of sociodemographic characteristics and neighbourhood-level social capital including all dynamic transitions from T1 to T2 compared with the healthy control group (*n* = 1395)

	Categories of the dependent variable^a^																					
	M1 (*n* = 233)		M2 (*n* = 92)		M3 (*n* = 119)		M4 (*n* = 55)		M5 (*n* = 19)		M6 (*n* = 84)		M7 (*n* = 39)		M8 (*n* = 67)		M9 (*n* = 15)		M10 (*n* = 13)		M11 (*n* = 44)	
	OR	*z*	OR	*z*	OR	*z*	OR	*z*	OR	*z*	OR	*z*	OR	*z*	OR	*z*	OR	*z*	OR	*z*	OR	*z*
Model	CI	*p*	CI	*p*	CI	*p*	CI	*p*	CI	*p*	CI	*p*	CI	*p*	CI	*p*	CI	*p*	CI	*p*	CI	*p*
1																						
Gender																						
Male	Ref		Ref		Ref		Ref		Ref		Ref		Ref		Ref		Ref		Ref		Ref	
Female	0.935	−0.46	1.818	2.47	0.830	−0.96	0.772	−0.93	1.515	0.83	1.276	1.02	0.900	−0.32	1.02	0.06	0.603	−0.97	1.64	0.81	0.823	−0.63
	0.70–1.25	0.647	0.13–2.92	**0.014**	0.57–1.21	0.338	0.45–1.33	0.353	0.57–4.02	0.404	0.80–2.03	0.306	0.47–1.72	0.751	0.61–1.68	0.955	0.22–1.68	0.334	0.50–5.37	0.417	0.45–1.51	0.530
Age																						
15–30	Ref		Ref		Ref		Ref		Ref		Ref		Ref		Ref		Ref		Ref		Ref	
31–45	0.580	−3.11	0.790	−0.91	0.600	−2.25	0.552	−1.78	0.470	−1.31	0.899	−0.40	0.507	−1.72	0.642	−1.55	0.579	−0.92	0.250	−2.07	0.684	−0.98
	0.41–0.82	**0.002**	0.48–1.31	0.360	0.38–0.94	**0.025**	0.29–1.06	0.076	0.15–1.46	0.191	0.53–1.52	0.689	0.23–1.10	0.085	0.37–1.13	0.122	0.18–1.85	0.355	0.07–0.93	**0.039**	0.32–1.46	0.327
46–65	0.579	−3.14	0.605	−1.83	0.512	−2.82	0.530	−1.90	0.561	−1.06	0.617	−1.66	0.491	−1.80	0.365	−3.02	0.329	−1.60	0.083	−2.36	0.811	−0.57
	0.41–0.81	**0.002**	0.35–1.04	0.067	0.32–0.82	**0.005**	0.28–1.02	0.057	0.19–1.63	0.289	0.35–1.09	0.097	0.23–1.06	0.071	0.19–0.70	**0.003**	0.08–1.28	0.109	0.11–0.66	**0.019**	0.40–1.67	0.570
2																						
ISC-variable	0.992	−0.04	1.572	1.42	1.403	1.17	1.532	1.05	1.543	0.65	1.020	0.06	1.331	0.59	0.647	−1.10	0.389	−1.12	0.528	−0.73	1.131	0.27
	0.64–1.55	0.971	0.84–2.94	0.156	0.80–2.47	0.242	0.69–3.39	0.292	0.42–5.70	0.515	0.52–2.01	0.954	0.52–3.42	0.553	0.30–1.41	0.272	0.08–2.02	0.261	0.09–2.96	0.468	0.46–2.81	0.791
3																						
SCT-variable	1.087	0.39	1.190	0.56	1.716	1.90	1.023	0.06	1.469	0.58	0.970	−0.10	1.324	0.60	0.914	−0.25	1.983	0.90	0.721	−0.43	1.487	0.89
	0.72–1.64	0.694	0.65–2.19	0.575	0.98–2.99	0.057	0.48–2.20	0.954	0.40–5.35	0.560	0.52–1.82	0.924	0.53–3.30	0.547	0.46–1.83	0.799	0.45–8.73	0.366	0.16–3.23	0.669	0.62–3.55	0.371
4																						
SocD-variable	0.769	−0.81	0.375	−2.25	0.501	−1.70	0.537	−1.08	0.769	−0.26	0.300	−2.75	0.469	−1.13	0.314	−2.36	0.366	−0.99	0.136	−2.12	0.511	−1.06
	0.41–1.46	0.420	0.47–1.29	0.331	0.23–1.11	0.089	0.17–1.67	0.282	0.11–5.54	0.794	0.13–0.71	**0.006**	0.13–1.74	0.257	0.12–0.82	**0.018**	0.05–2.69	0.324	0.02–0.86	**0.034**	0.15–1.77	0.289
5																						
Cogn-variable	0.769	−1.42	0.680	−1.40	0.672	−1.60	0.893	−0.34	1.361	0.59	0.974	−0.10	0.601	−1.21	1.017	0.06	0.860	−0.24	1.378	0.51	0.963	−0.10
	0.54–1.11	0.156	0.40–1.17	0.160	0.41–1.09	0.109	0.46–1.73	0.737	0.49–3.78	0.554	0.57–1.66	0.924	0.26–1.37	0.225	0.56–1.84	0.955	0.25–2.98	0.811	0.40–4.71	0.609	0.47–1.99	0.919

OR, odds ratio; CI, confidence interval, 95%.

ISC-variable, informal social control variable. Higher scores on the ISC-variable indicate lower levels of informal social control, meaning lower social capital.

SCT-variable, social control and trust variable. Higher scores on the SCT-variable indicate lower levels of social control and trust, meaning lower social capital.

SocD-variable, social disorganisation variable. Higher scores on the SocD-variable indicate lower levels of social disorganisation, meaning higher social capital.

Cogn-variable, cognitive social capital variable. Higher scores on the Cogn-variable indicate higher levels of cognitive social capital, meaning higher social capital.

The significant *p* values are bold.

a Categories of the dependent variable as presented in Fig. 1. All categories are compared to the reference category.

Participants living in neighbourhoods with higher scores on the SocD-variable, indicating lower levels of social disorganisation and thus higher social capital, were less likely to have emergent clinical PE (M6). Even if they did, their clinical PE were less likely to persist (M8). They were also less likely to transition to PD (M10). The other social capital variables were not associated with the transitions within the extended psychosis spectrum variable.

### Emergence, persistence and transition

Baseline sociodemographic characteristics and the level of social capital of the three outcome groups (emergence, persistence and transition) are summarised in Table 3. Lower levels of education and cannabis use were associated with all outcome groups. Family history of mental disorder was associated with persistence and transition. Lower age, not being married and being unemployed were associated with both emergence and transition. Current higher urbanicity, lower socioeconomic status, the presence of traumatic events and lower scores on the SocD-variable (meaning lower social capital) were associated with emergence.

**Table 3.** Comparison of baseline sociodemographic characteristics of the three outcome groups and the three reference groups

	Emergent		Non-emergent				Persistent		Non-persistent				Transition		Non-transition			
	*n* = 242		*n* = 1395				*n* = 119		*n* = 343				*n* = 28		*n* = 2103			
	*n*	%	*n*	%	χ^2^	*p*	*n*	%	*n*	%	χ^2^	*p*	*n*	%	*n*	%	χ^2^	*p*
Gender																		
Male	100	41.3	568	40.7	0.03	0.860	50	42.0	129	37.6	0.72	0.395	12	42.9	851	40.5	0.06	0.798
Female	142	58.7	827	59.3			69	58.0	214	62.4			16	57.1	1252	59.5		
Age																		
15–30	94	38.8	392	28.1	12.78	**0.002**	50	42.0	133	38.8	1.39	0.500	16	57.1	671	31.9	8.85	**0.012**
31–45	81	33.5	494	35.4			40	33.6	107	31.2			8	28.6	723	34.4		
46–65	67	27.7	509	36.5			29	24.4	103	30.0			4	14.3	709	33.7		
Education level																		
⩽8 years	169	69.8	799	57.3	13.46	**<0.001**	87	73.1	207	60.4	6.21	**0.013**	24	85.7	1265	60.2	7.55	**0.006**
>8 years	73	30.2	596	42.3			32	26.9	136	39.6			4	14.3	838	39.9		
Ethnicity																		
Turkish	183	75.6	1009	72.3	1.13	0.288	80	67.2	244	71.1	0.64	0.422	20	71.4	1519	72.2	0.01	0.925
Non-Turkish	59	24.4	386	27.7			39	32.8	99	28.9			8	28.6	584	27.8		
Marital status																		
Married	159	65.7	1048	75.1	9.45	**0.002**	70	58.8	218	63.6	0.84	0.359	14	50.0	1497	71.2	6.01	**0.014**
Not married	83	34.3	347	24.9			49	41.2	125	36.4			14	50.0	606	28.8		
Employment status																		
Unemployed	15	6.2	49	3.5	3.96	**0.047**	9	7.6	22	6.4	0.19	0.666	4	14.3	95	4.5	5.95	**0.015**
Not unemployed	227	93.8	1346	96.5			110	92.4	321	93.6			24	85.7	2008	95.5		
Cannabis use																		
User	13	5.4	16	1.2	21.15	**<0.001**	11	9.2	8	2.3	10.70	**0.001**	7	25.0	49	2.3	55.5	**<0.001**
Non-user	229	94.6	1379	98.9			108	90.8	335	97.7			21	75.0	2054	97.7		
Alcohol use																		
User	109	45.0	623	44.7	0.01	0.912	58	48.7	161	46.4	0.11	0.735	14	50	953	45.3	0.24	0.621
Non-user	133	54.7	772	55.3			61	51.3	182	53.1			14	50.0	1150	54.7		
Current urbanicity																		
Rural	6	2.5	34	2.4	19.50	**<0.001**	1	0.8	11	3.2	5.53	0.137	0	0.0	53	2.5	3.18	0.365
Low-urban	26	10.7	312	22.4			24	20.2	96	28.0			6	21.4	458	21.9		
Moderately urban	136	56.2	622	44.6			59	49.6	155	45.2			17	60.7	975	46.4		
Highly urban	74	30.6	427	30.6			35	29.4	81	23.6			5	17.9	617	29.3		
Urbanicity at birth																		
Village or district, population <50 000	122	50.4	719	51.5	0.96	0.811	57	47.9	144	42.0	6.23	0.101	13	46.4	1044	49.6	0.21	0.976
Population between 50 000 and 150 000	29	12.0	165	11.8			6	5.0	45	13.1			3	10.7	245	11.7		
Province with population >150 000	6	2.5	49	3.5			4	3.4	14	4.1			1	3.8	73	3.5		
District in metropolitan area	85	35.1	462	33.1			52	43.7	140	40.8			11	39.3	741	35.2		
Urbanicity between ages 6 and 15																		
Village or district, population <50 000	98	40.5	532	38.1	0.75	0.861	38	31.9	108	31.5	6.54	0.088	6	21.4	777	37.0	3.31	0.347
Population between 50 000 and 150 000	23	9.5	153	11.0			5	4.2	42	12.2			4	14.3	224	10.6		
Province with population >150 000	13	5.4	74	5.3			5	4.2	14	4.1			1	3.6	106	5.0		
District in metropolitan area	108	44.6	636	45.6			71	59.7	179	52.2			17	60.7	996	47.4		
Migration between ages 6 and 15																		
Migrated	25	10.3	175	12.5	0.94	0.332	19	16.0	36	10.5	2.5	0.112	4	14.3	256	12.2	0.12	0.734
Not migrated	217	89.7	1220	87.5			100	84.0	307	89.5			24	85.7	1847	87.8		
Socioeconomic status^a^																		
High employees	34	14.1	355	25.5	16.25	**0.001**	15	12.6	56	16.3	2.33	0.507	2	7.1	460	21.9	4.52	0.211
Skilled workers	65	26.9	368	26.9			31	26.1	101	29.5			11	39.3	566	26.9		
Small proprietors	46	19.0	213	15.3			20	16.8	58	16.9			4	14.3	338	16.1		
Manual workers	97	40.1	459	32.9			53	44.5	128	37.3			11	39.3	739	35.1		
Socioeconomic status at birth^a^																		
High employees	19	7.9	157	11.3	2.83	0.419	5	4.2	24	7.0	4.22	0.239	3	10.7	205	9.8	4.62	0.202
Skilled workers	40	16.5	241	17.3			20	16.8	73	21.3			4	14.3	374	17.8		
Small proprietors	95	39.3	509	36.5			43	36.1	131	38.2			6	21.4	780	37.1		
Manual workers	88	36.4	488	35.0			51	42.3	115	33.5			15	53.6	744	35.4		
Childhood adversity																		
None	204	84.3	1219	87.4	1.73	0.189	94	79.0	288	84.0	1.53	0.217	21	75.0	1807	85.9	2.7	0.100
At least one	38	15.7	176	12.6			25	21.0	55	16.0			7	25.0	296	14.1		
Traumatic events																		
None	140	57.9	945	68.4	10.33	**0.001**	52	43.7	183	53.4	3.30	0.069	14	50.0	1333	63.4	2.13	0.145
At least one	102	42.2	441	31.6			67	56.3	160	46.6			14	50.0	770	36.6		
Family history of mental disorder																		
None	195	80.6	1150	82.4	4.21	0.240	79	66.4	274	79.9	11.70	**0.008**	15	53.6	1702	80.9	15.71	0.001
Other/unknown	18	7.4	63	4.5			12	10.1	21	6.1			2	7.1	114	5.4		
Common mental disorder	25	10.3	148	10.6			25	21.0	36	10.5			9	32.1	234	11.1		
Severe mental disorder	4	1.7	34	2.4			3	2.5	12	3.5			2	7.1	53	2.5		
Neighbourhood-level	Mean	s.d.	Mean	s.d.	OR	*p*	Mean	s.d.	Mean	s.d.	OR	*p*	Mean	s.d.	Mean	s.d.	OR	*p*
ISC-variable	2.07	0.34	2.05	0.34	1.24	0.360	2.04	0.36	2.06	0.33	0.84	0.642	1.96	0.36	2.06	0.34	0.41	0.137
SCT-variable	2.32	0.37	2.29	0.37	1.33	0.191	2.29	0.33	2.30	0.34	0.93	0.852	2.31	0.40	2.30	0.36	1.07	0.907
SocD-variable	3.52	0.26	3.57	0.22	0.41	**0.006**	3.51	0.29	3.55	0.23	0.54	0.229	3.48	0.22	3.55	0.23	0.32	0.096
Cogn-variable	3.51	0.42	3.55	0.42	0.73	0.102	3.54	0.38	3.51	0.42	1.12	0.720	3.57	0.46	3.54	0.42	1.17	0.729

ISC-variable, informal social control variable. Higher scores on the ISC-variable indicate lower levels of informal social control, meaning lower social capital.

SCT-variable, social control and trust variable. Higher scores on the SCT-variable indicate lower levels of social control and trust, meaning lower social capital.

SocD-variable, social disorganisation variable. Higher scores on the SocD-variable indicate lower levels of social disorganisation, meaning higher social capital.

Cogn-variable, cognitive social capital variable. Higher scores on the Cogn-variable indicate higher levels of cognitive social capital, meaning higher social capital.

The significant *p* values are bold.

a Top to bottom: professional and non-manual high employee; non-manual low employee and skilled workers and technicians; small proprietors with or without employees; manual workers.

Mixed-effects logistic regression analysis revealed that participants who were living in neighbourhoods with lower scores on the SocD-variable were more likely to be in the emergent group compared with the non-emergent group (Table 4). The other social capital variables were not associated with any of the three outcome groups.

**Table 4.** Differential effect of baseline sociodemographic characteristics and neighbourhood-level social capital on the three outcome groups and the three reference groups

	Emergence				Persistence				Transition			
	OR	% 95 CI	*z*	*p*	OR	% 95 CI	*z*	*p*	OR	%95 CI	*z*	*p*
*Model 1*												
Gender												
Male	Ref	–			Ref	–			Ref	–		
Female	0.96	0.71–1.28	−0.31	0.757	0.74	0.45–1.22	−1.19	0.236	0.91	0.42–1.95	−0.25	0.804
Age												
15–30	Ref	–			Ref	–			Ref	–		
31–45	0.65	0.46–0.92	−2.47	**0.014**	1.08	0.61–1.90	0.26	0.794	0.47	0.20–1.10	−1.74	0.082
46–65	0.52	0.37–0.75	−3.56	**<0.001**	0.71	0.39–1.29	−1.13	0.260	0.24	0.08–0.73	−2.56	**0.010**
Parental SES	1.09	0.94–1.27	1.12	0.262	1.38	1.04–1.83	2.23	**0.026**	1.26	0.84–1.89	1.10	0.271
*Model 2*												
ISC-variable	1.31	0.82–2.07	1.14	0.256	0.91	0.42–1.98	−0.25	0.806	0.46	0.14–1.55	−1.25	0.210
*Model 3*												
SCT-variable	1.39	0.90–2.15	1.47	0.142	1.01	0.46–2.22	0.03	0.975	1.18	0.41–3.42	0.30	0.761
*Model 4*												
SocD-variable	0.39	0.21–0.74	−2.90	**0.004**	0.58	0.21–1.60	−1.05	0.294	0.30	0.08–1.15	−1.76	0.079
*Model 5*												
Cogn-variable	0.73	0.50–1.08	−1.57	0.116	1.11	0.57–2.13	0.30	0.765	1.17	0.49–2.83	0.36	0.721

OR, odds ratio; CI, confidence interval; SES, socioeconomic status. Lower SES compared with higher SES.

ISC-variable, informal social control variable. Higher scores on the ISC-variable indicate lower levels of informal social control, meaning lower social capital.

SCT-variable, social control and trust variable. Higher scores on the SCT-variable indicate lower levels of social control and trust, meaning lower social capital.

SocD-variable, social disorganisation variable. Higher scores on the SocD-variable indicate lower levels of social disorganisation, meaning higher social capital.

Cogn-variable, cognitive social capital variable. Higher scores on the Cogn-variable indicate higher levels of cognitive social capital, meaning higher social capital.

The significant *p* values are bold.

### Participants who moved to a different neighbourhood during the follow-up

Five hundred forty-five participants moved to a different neighbourhood between T1 and T2. Baseline social capital levels of those who moved were not significantly different from those who did not move. Five hundred and twenty of those who had moved had T2 social capital data (ISC-variable and SCT-variable). At T2, these 520 participants were living in a neighbourhood with significantly higher scores on the ISC-variable (meaning lower social capital) than the neighbourhood they were living in at the T1 assessment (2.28 ± 0.81; 2.06 ± 0.34; *t* = 5.61; *p* < 0.001). Mean scores on SCT-variable were not significantly different.

## Discussion

Conducted in a large community-based sample representative of the general population in a highly urbanised area of Turkey, TürkSch yielded comprehensive, high-quality data with a primary focus on the extended psychosis spectrum. In this longitudinal epidemiological survey of 2175 participants, we investigated whether neighbourhood social capital is associated with the risk of transitions within the extended psychosis spectrum over a 6-year follow-up period. The main finding of the primary analysis was that lower levels of social disorganisation decreased the risk of emergence and persistence of clinical PE and also the transition to PD. The finding that lower levels of social disorganisation decreased the risk of emergence was confirmed in our secondary analysis. The results, therefore, suggest that social disorganisation may be associated with transition towards the more severe end of the extended psychosis phenotype.

### Multinomial *v.* mixed-effects logistic regression analysis results

The multinomial regression showed significant associations between social disorganisation and emergent PE, persistent PE and transition to PD, whereas the mixed-effects regression showed a significant association between social disorganisation and emergent PE only. In the secondary analyses, the associations between the social disorganisation and the persistence of PE and transition to PD were not significant but in the same direction. The non-significance of the associations with persistence and transition in the secondary analysis may be related to the fact that multiple categories of the outcome measure were combined.

### Neighbourhood-level social capital

The results of the multinomial regression analyses showed that participants living in neighbourhoods with lower levels of social disorganisation were less likely to have emergent or persistent clinical PE and less likely to transition to PD. Even though the associations were in the same direction for all 11 groups, only three of them were statistically significant. Therefore, the overall association may not be very strong. The results of the mixed-effects regression analyses showed that the SocD-variable was only significantly associated with the emergence of PE.

There are no other longitudinal studies investigating social capital and the extended psychosis spectrum. Therefore, we could not compare our results directly. Within cross-sectional studies, there were only two that used social disorganisation as a measure of social capital (Binbay et al., 2012b; Kirkbride et al., 2008). One of them analysed the baseline data of TürkSch, and the authors showed that in neighbourhoods with more social disorganisation, respondents scored higher on the psychosis spectrum variable. They also reported that the association between the psychosis spectrum and severe mental illness in the family was stronger in disorganised neighbourhoods. However, the effect of social disorganisation was reducible to the other neighbourhood variables (Binbay et al., 2012b). Another study showed that social disorganisation was associated with schizophrenia incidence, but the association was not significant after considering social cohesion and trust (Kirkbride et al., 2008). Briefly, our findings are similar to those of previous studies, but contrary to their results, the current analyses revealed social disorganisation as the only social capital dimension significantly associated with the psychosis spectrum.

When we compare our results with those of other studies measuring various aspects of social capital, our finding is broadly consistent with several previous studies showing the protective effects of social capital in psychotic symptoms and disorders (Freeman et al., 2011; Kirkbride et al., 2007; Lofors & Sundquist, 2007; O'Donoghue et al., 2016). Kirkbride et al.'s finding that there is a U-shaped association between social capital and schizophrenia incidence (Kirkbride et al., 2008) is different from ours. A possible explanation may be the difference between the two measures of social capital. When analysing social cohesion and trust and schizophrenia, Kirkbride et al. showed a significant association, and we did not. Social cohesion and trust is a dimension of social capital, of which both lower and higher levels may increase the risk of psychosis expression. On the other hand, social disorganisation can be expected to have a one-way effect because the problems defined in the scale are more concrete and less open to interpretation. The causal mechanisms linking social disorganisation and psychosis expression are unclear. It is possible that neighbourhoods with higher levels of social disorganisation may facilitate or enhance factors related with psychosis such as immigration, traumatic events, cannabis use and air pollution (March et al., 2008).

Of all four social capital variables, we found significant results for only the SocD-variable; however, we did not find any significant results for the other three social capital variables. There may be several reasons for this pattern of results. First, the ISC-variable, SCT-variable and Cogn-variable were moderately to strongly correlated with each other, whereas the SocD-variable was not. An explanation for this might be that the SocD-variable represents a different dimension of social capital. The ISC-variable, SCT-variable and Cogn-variable may represent a more cognitive aspect of social capital that is called ‘collective efficacy’ (Sampson, 1997), whereas the SocD-variable may represent more structural aspects such as concentrated disadvantage and population density (McCulloch, 2003). Second, various aspects of social capital may be more important in different contexts. In Izmir, social disorganisation may represent a more robust effect of social capital on psychosis (Saraçoǧlu & Demirtaş-Milz, 2014) given very large differences between neighbourhoods in concentrated disadvantage and population density (Sönmez, 2007; Metropolitan Municipality of İzmir, 2007). Finally, social disorganisation may more powerfully represent the social contextual change in Turkey between T1 and T2 because of the socioeconomic impact of the 2008 financial crisis (2010–2014) (Aytaç, Rankin, & İbikoğlu, 2015).

### Demographic and background characteristics

In general, the findings are in line with the literature (Linscott & Van Os, 2013; Van Os & Reininghaus, 2016). However, it is noteworthy that we did not find ethnicity to be a significant risk factor for psychosis. A plausible explanation for this might be that the risk factor is not being a minority *per se* but rather occupying a minority position in society (Van Os, Kenis, & Rutten, 2010). The minority groups investigated in our study do not stand out in relation to the general population in terms of easily distinguishable traits (e.g. skin colour, religious practices).

### Methodological issues

Our findings should be considered in light of several limitations. First of all, a limitation is the possibility of selection bias caused by attrition over time. However, the attrition analysis revealed very small effect sizes; details have been published elsewhere (Kırlı et al., 2019). Second, around a quarter of the participants had moved to neighbourhoods with lower levels of social capital between baseline and follow-up. Since it is not known how long it takes for environmental factors to cause changes in mental health, we chose to use the baseline social capital data in our analyses. In addition, the follow-up social capital data were collected from the participants themselves and thus, the follow-up social capital data could be biased by the mental health status of the participants. Since we found no significant difference between the baseline neighbourhood-level social capital data of those who moved and those who did not move, it is unlikely that the mobility of the participants might have affected our results. Third, psychotic patients may tend to neglect or underreport their symptoms. In order to minimise this bias, additional information was gathered through clinical interviews and health records. Fourth, we defined the extended psychosis spectrum categories based on the presence of positive symptoms and the clinical impairment they cause. This definition is arbitrary, and the spectrum could be constructed in different ways. However, the sensitivity of CIDI is high for delusions and hallucinations, and this increased the clinical validity of our spectrum. As a result, our results cannot be generalised to negative or disorganised dimensions of psychosis. Finally, some of the questions used in the social disorganisation scale rely on subjective moral values, such that the results of this investigation can not be generalised to every culture.

This is the first longitudinal community-based study investigating whether social capital is associated with the risk of transitions within the extended psychosis spectrum over a 6-year follow-up period. The strengths of our study are having high-quality data of a large number of participants at two different time points and the data being collected by house visits by trained interviewers. The longitudinal design allowed us to create categories based on dynamic transitions within the extended psychosis spectrum, and this approach is more useful than working with categories based on cross-sectional data. Working with a general population sample was advantageous because we were more likely to include individuals who might not be captured in a clinical sample. The fact that data were collected randomly throughout a geographical area prevented help-seeking bias, thus revealing the spectrum, including subclinical manifestations. Another strength is that the neighbourhood-level variables were independently collected from a different sample. In addition to this, social capital was measured directly, whereas, in many other studies, proxy measurements such as voter turnout were implemented.

## Conclusion

In conclusion, we found that lower levels of social disorganisation decreased the risk of emergence and persistence of clinical PE and also the transition to PD. The evidence about emergence was particularly robust. Our findings implicate that one dimension of social capital may reduce the risk of psychosis expression. Whilst replication of this finding is required, it may point to targeting the levels of social disorganisation as a public health measure associated with population psychosis risk.
